# Psychometric evaluation of the Japanese Edinburgh Postnatal Depression Scale for screening postpartum anxiety

**DOI:** 10.3389/fpsyt.2025.1659497

**Published:** 2025-09-24

**Authors:** Ekachaeryanti Zain, Yuichiro Watanabe, Shinpei Takabayashi, Leakhena Por, Saori Fujita, Sachie Moriyama, Aiko Honma, Naoki Fukui, Shuken Boku

**Affiliations:** ^1^ Department of Psychiatry, Niigata University Graduate School of Medical and Dental Sciences, Niigata, Japan; ^2^ Department of Psychiatry, Uonuma Kikan Hospital, Niigata, Japan; ^3^ Faculty of Medicine, Niigata University School of Medicine, Niigata, Japan; ^4^ Department of Nursing, Niigata University Medical and Dental Hospital, Niigata, Japan; ^5^ Medical Education Center, Faculty of Medicine, Niigata University, Niigata, Japan

**Keywords:** Edinburgh Postnatal Depression Scale, anxiety, postpartum women, psychometrics, ROC analysis

## Abstract

**Background:**

Despite the acknowledged importance of addressing postpartum anxiety alongside postpartum depression, standardized screening tools specifically developed for this purpose remain limited.

**Objective:**

This study aimed to validate the anxiety factor of the Edinburgh Postnatal Depression Scale (EPDS) and to determine optimal cutoff scores for screening postpartum anxiety.

**Methods:**

EPDS and the State-Trait Anxiety Inventory (STAI) were collected from 100 Japanese women at one month postpartum at Niigata University Medical and Dental Hospital between May 18, 2021, and December 28, 2022, using random convenience and purposive sampling. Confirmatory factor analysis (CFA) was conducted on data from 84 participants to test six previously proposed EPDS factor models, and receiver operating characteristic (ROC) analysis was performed on data from 83 participants to determine area under the curve (AUC) values and cutoff scores. The EPDS anxiety subscales with three items (EPDS-3A) and four items (EPDS-4A) were separately evaluated as predictor variables, with STAI state and trait anxiety as criterion measures. We assessed accuracy, sensitivity, and specificity.

**Results:**

All models with two-factor and three-factor showed a good fit to the data, with two models with EPDS-3A being superior among other models (comparative fit index = 1.000, root mean square error of approximation = 0.001). ROC analyses indicated good testing accuracy of the EPDS anxiety subscales for detecting both state and trait anxiety. For EPDS-3A, the AUCs were 0.832 (95% CI 0.735–0.930) for state anxiety with an optimal cutoff of ≥3 (sensitivity 79.2%, specificity 79.7%), and 0.912 (95% CI 0.837–0.988) for trait anxiety with an optimal cutoff of ≥4 (sensitivity 82.4%, specificity 84.8%). For EPDS-4A, the AUCs were 0.833 (95% CI 0.736–0.930) for state anxiety with an optimal cutoff of ≥4 (sensitivity 79.2%, specificity 71.2%), and 0.935 (95% CI 0.867–1.000) for trait anxiety with an optimal cutoff of ≥5 (sensitivity 88.2%, specificity 87.9%).

**Conclusion:**

Both the EPDS-3A and EPDS-4A demonstrated good model fit and screening accuracy for anxiety at one month postpartum. Integrating anxiety screening into routine postpartum care may improve maternal mental health outcomes. Future studies involving clinical settings and larger cohort studies are recommended to improve external validity.

## 1 Introduction

The perinatal period, encompassing pregnancy and the first year postpartum, is a critical time marked by an increased risk of psychological distress and mood disturbances in women (1, 2). Women experience several types of mental disorders including mood disorders, anxiety disorders, obsessive compulsive disorder, psychosis, eating disorders, and substance use disorders (3). Depression and anxiety disorders are particularly prevalent during this period and frequently co-occur (4). However, postpartum anxiety is underdiagnosed despite its high prevalence and clinical significance (1). A recent meta-analysis estimated that 20.7% of perinatal women experience anxiety symptoms, suggesting that perinatal anxiety may be more common than previously recognized (5). Global prevalence estimates of perinatal anxiety range from 15% to 23% (5, 6), slightly exceeding perinatal depression at approximately 17% worldwide (7). A similar prevalence pattern was reflected in Japan, with postpartum anxiety rates of 18.5%–35.4% compared to postpartum depression at about 14.3% (8). Although the EPDS is widely used in Japan as a routine screening tool for pregnant women requiring social and mental support (9), screening for anxiety has not yet been specifically implemented in clinical practice.

While it is common to experience some anxiety during the transition to caring for a newborn, such symptoms are typically temporary and should not impair daily functioning (10). Although postpartum anxiety is not yet clearly defined as a distinct clinical entity, one study reported that symptoms of generalized anxiety disorder occurring within four weeks after childbirth may reflect postpartum anxiety (10). The symptoms may include excessive anxiety and worry with physical symptoms such as restlessness, fatigue, irritability, difficulty concentrating, muscle tension, and sleep disturbances, which can significantly interfere with daily life functioning (11). However, postpartum women were more likely to worry about their parenting abilities and others’ judgments (12).

Studies have consistently shown that perinatal anxiety is associated with adverse outcomes, including pregnancy and childbirth complications, negative infant health effects, and an increased risk of maternal suicide (13, 14). Mothers with early postpartum anxiety less than 3 months after delivery were more likely to develop postpartum depression than those of 6 months postpartum or later (15). Moreover, postpartum anxiety may negatively impact mother–infant bonding, cause no initial breastfeeding at 3 weeks postpartum, contribute to adverse emotional outcomes in children, and be associated with delayed mental development in children (16–19). These findings underscore the significant impact of perinatal anxiety on both maternal mental health and child development, reinforcing the need for increased awareness and early intervention efforts.

Despite the recognized importance of addressing perinatal anxiety, standardized screening tools specifically designed for this purpose remain underdeveloped. Many healthcare providers continue to rely on instruments originally intended to screen for perinatal depression. Among these, the Edinburgh Postnatal Depression Scale (EPDS) is one of the most widely used tools for detecting postpartum depression and has been validated in multiple languages (20, 21), including Japanese (22). The EPDS has demonstrated strong psychometric properties across diverse populations and effectively identifies postpartum depression, with factor analyses revealing subscales related to depression, anhedonia, and anxiety factors (23, 24).

Previous studies have examined the use of the three-item anxiety subscale of the EPDS (EPDS-3A; items 3, 4, and 5) as a potential screening tool for perinatal anxiety, with some suggesting cutoff scores of ≥4 for antenatal and ≥5 for postpartum women (1, 25–27). However, many of these studies relied on anxiety indicators derived from demographic questionnaires rather than standardized anxiety measures (26, 27), limiting the ability to rigorously assess the predictive validity of the EPDS-3A. Although the EPDS is considered reliable during the first six months postpartum, the four-item anxiety subscale (items 3, 4, 5, 10) derived from a community-based dataset showed limited utility when compared with the six-item short form of the Spielberger State-Trait Anxiety Inventory (STAI) (28, 29). In response to these limitations, Japanese validation studies (24, 30) identified an alternative four-item anxiety subscale (EPDS-4A; items 3, 4, 5, and 6) that demonstrated superior model fit within a three-factor EPDS structure (24). However, the acceptability and effectiveness of EPDS-4A compared to widely studied EPDS-3A as a screening tool for perinatal anxiety and the cutoff scores for both subscales in Japanese populations have yet to be confirmed.

Building on previous findings, the present study is the first study to validate the Japanese version of the EPDS-4A as a screening tool for anxiety in women at one-month postpartum, while also evaluating the psychometric performance of the widely studied EPDS-3A. The one-month postpartum period has been identified as a period of increased risk for the onset of psychological symptoms, with women who have preexisting mental health conditions exhibiting a substantial increase in psychiatric episodes during this period, particularly within the first month following delivery (31, 32). To our knowledge, this is the first study to validate and confirm the utility of EPDS-3A and EPDS-4A using a standardized anxiety scale (STAI) among Japanese women at one month postpartum. This will be achieved by testing the six previously proposed EPDS factor models: the three-factor model with EPDS-4A from Matsumura et al. (24); the three-factor models with EPDS-3A from Kubota et al. (30) and Lautarescu et al. (25); and the two-factor models with EPDS-3A from Matthey (33), Swalm et al. (27), and Smith-Nielsen et al. (26). If a good model fit is established, cutoff scores for the EPDS anxiety subscales will be determined by examining their correlations with anxiety levels measured using the STAI, assessing both state and trait anxiety (34, 35). By enabling the EPDS anxiety subscale to reliably detect postpartum anxiety during routine depression screening in the Japanese context, this study intends to support early detection and intervention, ultimately improving health outcomes for both Japanese mothers and their children.

## 2 Materials and methods

### 2.1 Participants

This study used data on the EPDS and the STAI collected at one month postpartum as part of the “Labor Pain and Perinatal Mental Health” project. This study was previously conducted on convenience and purposive samples that were randomly drawn. The dataset comprised responses from 100 healthy Japanese women aged 18 years or older who had a singleton pregnancy and delivered vaginally between 37 and 41 weeks of gestation at Niigata University Medical and Dental Hospital, Niigata City, Japan. Participants were asked to fill out a self-report questionnaire. A total of 84 participants completed the EPDS with no missing responses, and of those, 83 also completed the STAI without missing any items. Data were collected between May 18, 2021, and December 28, 2022, during the coronavirus disease 2019 (COVID-19) pandemic in Japan (March 6, 2020, to April 21, 2023). Exclusion criteria included serious physical complications, significant pregnancy-related complications, or severe psychiatric disorders as reported from the medical record, such as schizophrenia or major depressive disorder. The exclusion of women with severe psychiatric disorders was intended to focus on the general postpartum population and to examine the utility of the EPDS anxiety subscale as a screening tool in non-clinical settings, where early detection is most relevant before psychiatric diagnosis is established.

### 2.2 Measures

The measures used in this study were merely self-report questionnaires of EPDS and STAI without clinician diagnostic confirmation, administered using separate printed paper forms. The item order was consistent with the previously validated Japanese versions of the original instruments. The forms were compiled into a single questionnaire set, presented in the following sequence: the EPDS followed by the STAI. It was therefore assumed that most participants completed the questionnaires in the order provided.

#### 2.2.1 Edinburgh Postnatal Depression Scale

The EPDS is a 10-item self-administered questionnaire developed to screen for postpartum depression (20). Each item (e.g., “I have been able to laugh and see the funny side of things”, “I have been anxious or worried for no good reason”, and “I have been so unhappy that I have had difficulty sleeping”) is rated on a four-point Likert scale, with responses scored from 0 to 3 based on how often the respondent experienced each item over the past seven days. Total scores range from 0 to 30, with higher scores indicating greater risk of developing depressive disorder. The EPDS is widely used in clinical and research settings and has been translated into more than 60 languages (36, 37).

In this study, we use the Japanese version of the EPDS, developed by Okano et al. (22) using a back-translation method. This version has demonstrated strong psychometric properties, including good internal consistency (Cronbach’s α = 0.78), excellent test-retest reliability (r = 0.92), and an optimal cut-off score of 8/9, yielding 75% sensitivity and 93% specificity. Previous research has examined the factor structure of the EPDS (23, 38), and the Japanese version is considered to follow a three-factor model comprising anxiety (items 3, 4, 5, and 6), depression (items 7, 9, and 10), and anhedonia (items 1 and 2), which demonstrated acceptable goodness-of-fit and temporal stability (24).

Based on the good-fitting model of the Japanese version of the EPDS identified in this study, the EPDS-4A (items 3, 4, 5, and 6) was evaluated, in comparison with EPDS-3A (items 3, 4, 5), for screening postpartum anxiety in Japanese women.

#### 2.2.2 State-trait anxiety inventory

The STAI is a widely used measure of individual differences in anxiety (35). It consists of two 20-item subscales: the state-anxiety subscale assesses the intensity of anxiety experienced in the present moment (e.g., “I am tense; I am worried” and “I feel calm; I feel secure”), while the trait-anxiety subscale measures the general tendency to experience anxiety (e.g., “I worry too much over something that really doesn’t matter” and “I am content; I am a steady person”). Items are rated on a 4-point Likert scale ranging from 1 (“not at all”) to 4 (“very much so”), with total scores for each subscale ranging from 20 to 80.

The STAI has demonstrated strong validity and reliability (35, 37, 39), including its Japanese version (34, 40). The Japanese version shows high internal consistency, with Cronbach’s α values of 0.92 for state anxiety and 0.89 and 0.71 for trait anxiety at one-hour and three-month intervals, respectively (40).

Based on previous Japanese studies involving general student populations and clinical samples (anxious patients were administered diazepam before surgical procedures), cutoff scores of ≥ 42 for state anxiety and ≥ 45 for trait anxiety have been suggested for female participants (34, 40, 41). Therefore, in the present study, these cutoff values were used to define the presence of state and trait anxiety, although future studies may refine thresholds specific to postpartum cohorts.

### 2.3 Statistical analyses

#### 2.3.1 Descriptive statistics

Descriptive statistics, including means and standard deviations, were calculated for each factor of the EPDS and STAI scores.

#### 2.3.2 Confirmatory factor analysis

Confirmatory factor analysis (CFA) was conducted to compare six models of the EPDS. The six models were: the three-factor model with EPDS-4A from Matsumura et al. (24), the three-factor models with EPDS-3A from Kubota et al. (30) and Lautarescu et al. (25), and the two-factor models with EPDS-3A from Matthey (33), Swalm et al. (27), and Smith-Nielsen et al. (26). A sample size justification was based on the rule of thumb that recommended a ratio of 5–10 participants per estimated parameter. With 12 parameters in the model, a sample size of 84 participants was considered adequate. The reverse-scored items were retained as the original responses in conducting the CFA, preserving the natural covariance structure among items. Model fit was determined as a good fit using the comparative fit index (CFI ≥ 0.90) and the root mean square error of approximation (RMSEA ≤ 0.08) (42). CFA was needed to confirm structural validity prior to performing receiver operating characteristic curve (ROC) analysis.

#### 2.3.3 Receiver operating characteristic analysis

The ROC analysis was conducted to evaluate the diagnostic accuracy of the EPDS-3A and EPDS-4A in predicting state anxiety and trait anxiety, as measured by the STAI. To detect an AUC of 0.85 and effects of 0.15 with a 95% confidence level and 80% power, a minimum of 70 sample size is required (43). Our sample of 83 participants with complete EPDS and STAI data meets this threshold and is adequate for preliminary diagnostic validation. This study did not include cross-validation in an independent sample, which limits the ability to confirm the stability and generalizability of the factor structure.

ROC analyses were performed separately for the EPDS-3A and EPDS-4A. In each analysis, the total score of the respective subscale served as the independent variable, while state and trait anxiety were dependent variables, dichotomized using cutoff scores of ≥ 42 and ≥ 45, respectively. The discriminatory power of each subscale was evaluated by calculating the area under the curve (AUC), interpreted as follows: 0.5 = no discrimination, 0.7–0.8 = acceptable, 0.8–0.9 = good, and >0.9 = excellent. The standard error of the AUC, 95% confidence interval (CI), and P-values were reported to evaluate precision and statistical significance. A significance threshold of *P* < 0.025 was applied using the Bonferroni correction. Optimal cutoff scores for the EPDS-3A and EPDS-4A were determined separately using the maximum value of the Youden index.

#### 2.3.4 Correlation analysis

Correlation analysis with Pearson’s correlation was conducted to explore the relationships between anxiety and other psychological dimensions identified within the EPDS, which were depression and anhedonia. It is to provide insight into the clinical use of the EPDS as a multidimensional screening tool for anxiety and depression. We hypothesized that the EPDS-4A scores yielded in the Japanese population (24) would show a moderate to strong positive correlation with other subscales of EPDS.

All statistical analyses were performed using the Statistical Package for the Social Sciences (SPSS) version 31 (IBM Corp., Armonk, NY, USA) and Amos version 25.0.0 (IBM Japan, Tokyo, Japan).

## 3 Results

### 3.1 Descriptive statistics

Data from 100 postpartum women were included in the analysis. The mean age was 34.3 ± 4.8 years; 47 participants were primiparous and 53 were multiparous. Among 100 participants, 84 participants completed the EPDS without any missing data, and among them, 83 also completed the STAI in full at one month postpartum (**Table 1**).

**Table 1:** EPDS and STAI scores of participants at one month postpartum with EPDS-4A.

Variables	Scores
EPDS (n = 84)	
Anxiety	3.23 ± 2.31
Depression	0.51 ± 0.96
Anhedonia	0.23 ± 0.62
Total Score	4.46 ± 3.89
STAI (n = 83)	
State Anxiety	40.0 ± 9.39
Trait Anxiety	36.5 ± 9.62
Total Score	73.5 ± 18.1

Data are presented as mean ± standard deviation.

EPDS, Edinburgh Postnatal Depression Scale; STAI, State-Trait Anxiety Inventory.

### 3.2 Confirmatory factor analysis

**Figure 1:**
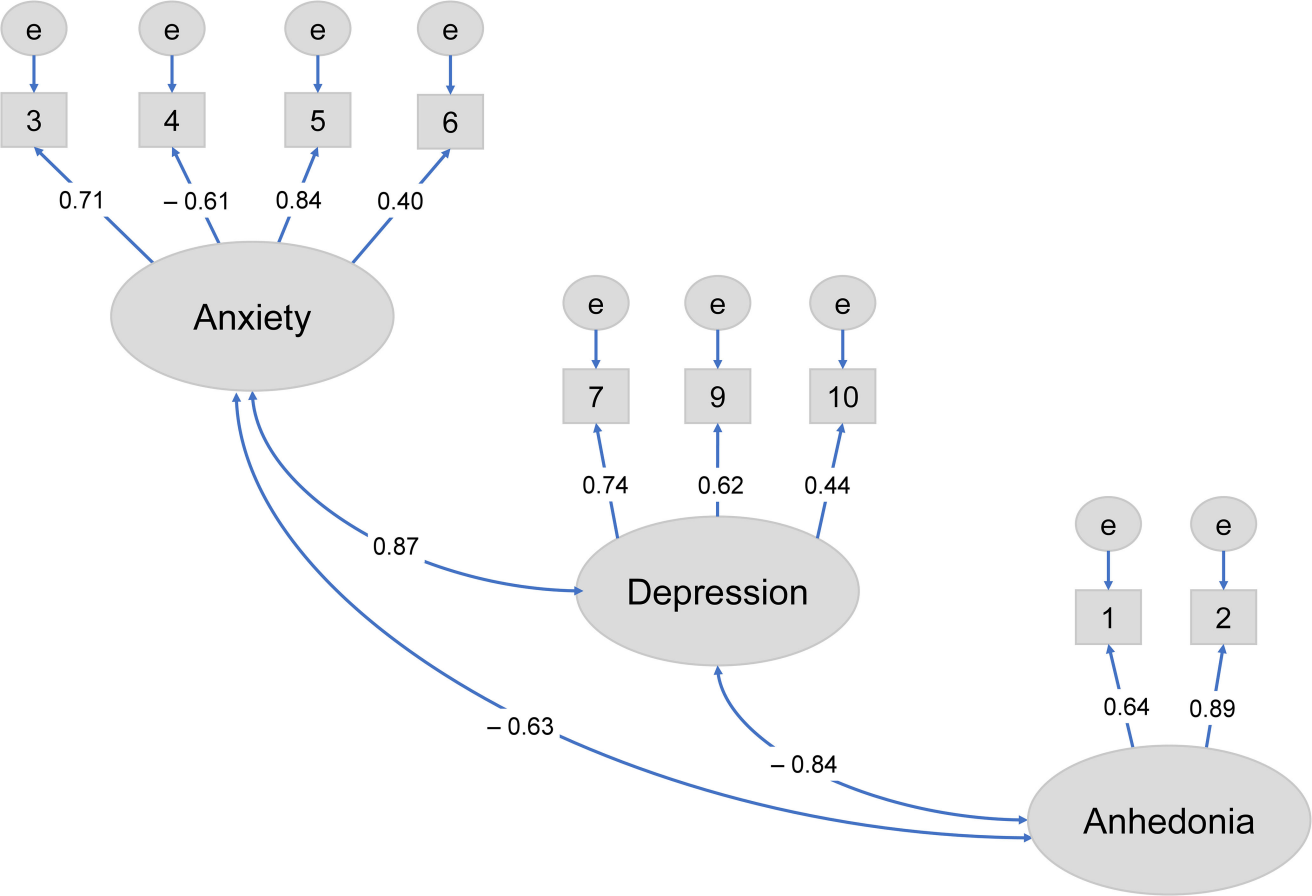
Confirmatory factor analysis of the three-factor model of the Edinburgh Postnatal Depression Scale with EPDS-4A (24) at one month postpartum.

CFA was conducted using data from 84 participants without missing values. CFA showed that the three-factor structure model of the EPDS-4A from Matsumura et al. (24) provided a good fit to the data at one month postpartum (CFI = 0.982, RMSEA = 0.040) (**Figure 1**; **Table 2**). Based on this model, items 3, 4, 5, and 6 were extracted and designated as the EPDS-4A. Therefore, EPDS-4A validity was confirmed.

**Table 2:** Confirmatory factor analysis of six models from previous studies (n = 84).

Model	Study	Country	Factors	Items	CFI	RMSEA
3-factor model with EPDS-4A	Matsumura et al. (24)	Japan	Anxiety	3, 4, 5, 6	0.982	0.040
			Depression	7, 9, 10		
			Anhedonia	1, 2		
3-factor model with EPDS-3A	Kubota et al. (30)	Japan	Anxiety	3, 4, 5	1.000	0.001
			Anhedonia	1, 2		
			Depression	7, 8, 9		
	Lautarescu et al. (25)	United Kingdom	Anxiety	3, 4, 5	0.997	0.018
			Depression	7, 8, 9, 10		
			Anhedonia	1, 2		
2-factor model with EPDS-3A	Matthey (33)	Australia	Anxiety	3, 4, 5	0.976	0.045
			Depression	1, 2, 6, 7, 8, 9, 10		
	Swalm et al. (27)	Australia	Anxiety	3, 4, 5	1.000	0.001
			Anhedonia	1, 2		
	Smith-Nielsen et al. (26)	Denmark	Anxiety	3, 4, 5	0.995	0.027
			Depression	1, 2, 8, 9		

All models demonstrated a good fit.

EPDS-4A, Edinburgh Postnatal Depression Scale with the four-item anxiety subscale; EPDS-3A, Edinburgh Postnatal Depression Scale with the three-item anxiety subscale; CFI, comparative fit index; RMSEA, root mean square error of approximation.

We also conducted CFA to compare the factor models proposed in six previous studies (**Table 2**). The results indicated that all models demonstrated good fit to the data: the three-factor models with EPDS-3A from Kubota et al. (30) and Lautarescu et al. (25) with CFI = 1.000 and 0.997, RMSEA = 0.001 and 0.018, respectively; and the two-factor models with EPDS-3A from Matthey (33), Swalm et al. (27), and Smith-Nielsen et al. (26) with CFI = 0.976, 1.000, and 0.995, RMSEA = 0.045, 0.001, and 0.027, respectively. Among these, the EPDS-3A with three-factor (30) and the two-factor models (27) provided the best fit to our data.

### 3.3 Receiver operating characteristic analysis

**Figure 2:**
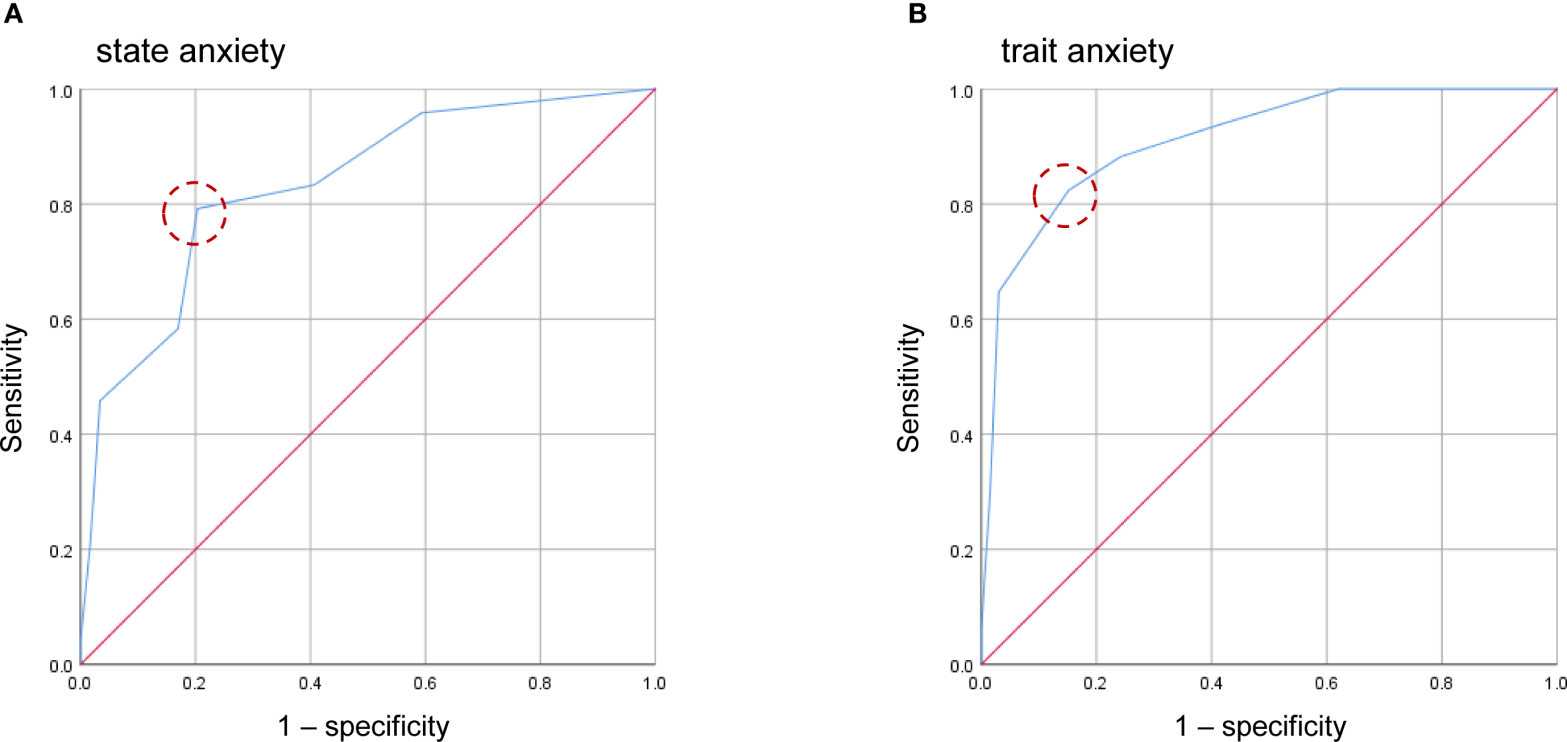
Receiver operating characteristic (ROC) curves of the four-item anxiety subscale of the EPDS-3A for state anxiety **(A)** and trait anxiety **(B)**. Dashed circles indicate the optimal cutoff points estimated using the maximum value of the Youden index.

The ROC was conducted using data from 83 participants without missing values. The EPDS-3A showed good and excellent discrimination for state anxiety and trait anxiety, respectively. The AUC for the EPDS-3A score was 0.832 (95% CI 0.735–0.930) for state anxiety and 0.912 (95% CI 0.837–0.988) for trait anxiety (**Figure 2**), both statistically significant (P < 0.001). The optimal cutoff for state anxiety was ≥ 3 (cutoff point 2/3), yielding a sensitivity of 79.2% and a specificity of 79.7%. For trait anxiety, the optimal cutoff was ≥ 4 (cutoff point 3/4), with a sensitivity of 82.4% and specificity of 84.8% (**Table 3**).

**Table 3:** Sensitivity, specificity, and Youden index of each cutoff score of the EPDS-3A in identifying state and trait anxiety (n = 83).

Cutoff score	State anxiety			Trait anxiety		
	Sensitivity	Specificity	Youden index	Sensitivity	Specificity	Youden index
1/2	0.833	0.593	0.426	0.941	0.576	0.517
**2/3**	**0.792**	**0.797**	**0.589**	0.882	0.758	0.640
**3/4**	0.583	0.831	0.414	**0.824**	**0.848**	**0.672**
4/5	0.458	0.966	0.424	0.647	0.970	0.617
5/6	0.208	0.983	0.191	0.294	0.985	0.279

Bold values indicate the cutoff scores with the maximum Youden index.

EPDS-3A, Edinburgh Postnatal Depression Scale with the three-item anxiety subscale.

**Figure 3:**
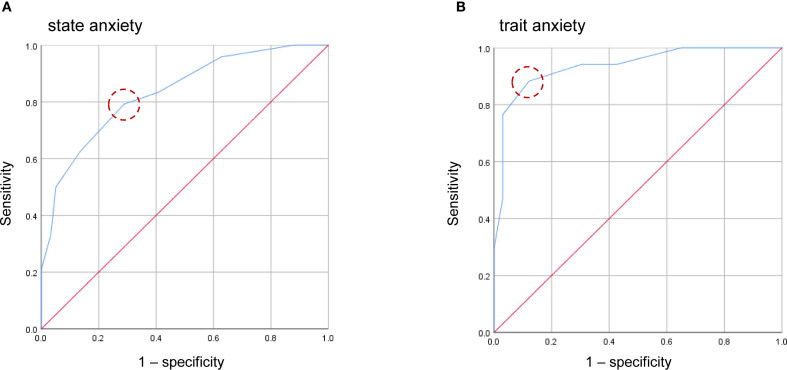
Receiver operating characteristic (ROC) curves of the four-item anxiety subscale of the EPDS-4A for state anxiety **(A)** and trait anxiety **(B)**. Dashed circles indicate the optimal cutoff points estimated using the maximum value of the Youden index.

The EPDS-4A also showed good and excellent discrimination for state anxiety and trait anxiety, respectively. The AUC for the EPDS-4A score was 0.833 (95% CI 0.736–0.930) for state anxiety and 0.935 (95% CI 0.867–1.000) for trait anxiety (**Figure 3**), both statistically significant (*P* < 0.001). The optimal cutoff for state anxiety was ≥ 4 (cutoff point 3/4), yielding a sensitivity of 79.2% and a specificity of 71.2%. For trait anxiety, the optimal cutoff was ≥ 5 (cutoff point 4/5), with a sensitivity of 88.2% and specificity of 87.9% (**Table 4**).

**Table 4:** Sensitivity, specificity, and Youden index of each cutoff score of the EPDS-4A in identifying state and trait anxiety (n = 83).

Cutoff score	State anxiety			Trait anxiety		
	Sensitivity	Specificity	Youden index	Sensitivity	Specificity	Youden index
2/3	0.833	0.593	0.426	0.941	0.576	0.517
**3/4**	**0.792**	**0.712**	**0.504**	0.941	0.697	0.638
**4/5**	0.625	0.864	0.489	**0.882**	**0.879**	**0.761**
5/6	0.500	0.949	0.449	0.765	0.97	0.735
6/7	0.333	0.966	0.299	0.471	0.97	0.441

Bold values indicate the cutoff scores with the maximum Youden index.

EPDS-4A, Edinburgh Postnatal Depression Scale with the four-item anxiety subscale.

The AUC > 0.9 for trait anxiety suggests EPDS-3A and EPDS-4A may be a highly accurate tool for postpartum anxiety screening.

### 3.4 Correlation analysis

We conducted a Pearson’s correlation analysis among the three factors with EPDS-4A identified through CFA. The anxiety factor showed a significant positive correlation with both depression (*r* = 0.691, *P* = 0.0001) and anhedonia (*r* = 0.501, *P* = 0.0001), indicating positive correlations among these dimensions.

## 4 Discussion

This study validated the three-factor structure of the Japanese version of the EPDS at one month postpartum and determined cutoff scores for its anxiety subscale (EPDS-4A) in comparison with the other three- and two-factor structures of EPDS-3A for use in screening postpartum anxiety among Japanese women.

Our CFA results indicated that all six previously proposed models with either EPDS-3A or EPDS-4A demonstrated good fit. These findings support the three-factor structure of EPDS, consisting of depression, anhedonia, and anxiety factors, consistent with previous studies that identified similar structures in Japanese and British women (24, 25, 30). However, differences were observed in the composition of the anxiety factor. The Japanese version includes items 3, 4, 5, and 6 (24), whereas the other Japanese version and British version (25, 30) identify items 3, 4, and 5 (EPDS-3A). Studies conducted among Australian and Danish women have also validated the EPDS-3A based on a two-factor structure (26, 27). Although the EPDS-3A has been supported in various populations, including Japan (30), evidence from a recent large-scale study involving 91,063 Japanese women found that the model incorporating the EPDS-4A provided a superior fit compared to earlier models (24). That study reported that three-factor structures generally showed better goodness-of-fit indices compared to two-factor structures, especially when the anxiety factor included four items (items 3, 4, 5, and 6) (24). The current study builds on these findings and further confirms the suitability of the three-factor structure with EPDS-4A in a population of Japanese postpartum women, equal to those with EPDS-3A.

Differences in EPDS factor structures across populations may result in varying outcomes, particularly in the anxiety subscale, depending on population characteristics. These differences may also reflect cultural and linguistic influences specific to each country, including Japan (24, 44). While the EPDS-3A has been studied across multiple countries and demonstrated cross-cultural validity (25–27, 30, 33), the EPDS-4A emerged as a novel finding in the Japanese population (24). Matsumura et al. (24) discussed that Japanese women may be more likely to report physical rather than psychological symptoms of anxiety, consistent with cultural norms emphasizing indirect emotional expression. Given that the EPDS does not contain items assessing somatic symptoms, its sensitivity in detecting anxiety among this population may be limited. Specifically, items 3 (“*I have blamed myself unnecessarily when things went wrong*”), 4 (“*I have been anxious or worried for no good reason*”), and 5 (“*I have felt scared or panicky for no very good reason*”) alone might not sufficiently capture the anxiety construct in statistical analyses. However, including item 6 (“*Things have been getting on top of me*”) may provide additional insight, better capturing anxiety-related experiences and thus enhancing the validity of the anxiety factor among the Japanese population. Although cultural factors may influence the reporting of postpartum anxiety symptoms, these interpretations were not directly examined in the present dataset. Future research should incorporate culturally comparative designs to more rigorously investigate the cross-cultural validity of EPDS-4A.

Derived from the three-factor structure, the present study offers a novel contribution by demonstrating that the EPDS-4A can be used to screen for anxiety at one month postpartum. Previous research has primarily supported the EPDS-3A across diverse perinatal populations. For example, an Australian study confirmed its effectiveness among antenatal women (27), while a Danish study validated its use in both antenatal and postnatal populations (26). Additionally, studies in England and Northern Ireland identified its applicability for postnatal anxiety at three months postpartum (45), and another study in Australia validated it at six weeks postpartum (33). In contrast to our study, which used the STAI as a standardized measure of anxiety, these studies employed a range of tools, including anxiety-related items from demographic questionnaires (27), the Hopkins Symptom Check-List (26), self-identified anxiety (45), and the Diagnostic and Statistical Manual of Mental Disorders, Third Edition-Revised and Fourth Edition (DSM-III-R and DSM-IV) (33, 46). While earlier research has focused exclusively on the EPDS-3A, this is the first study to confirm the utility of the EPDS-4A (items 3, 4, 5, and 6) as a screening tool for postpartum anxiety. Therefore, in addition to the above-mentioned cultural influences, variations in the EPDS factor structures across studies may also reflect methodological differences and the choice of criterion measures used to assess anxiety.

The ROC analysis further confirmed the discriminatory power of the EPDS-3A and EPDS-4A in predicting both state and trait anxiety. For the EPDS-3A, the AUC was 0.832 for state anxiety and 0.912 for trait anxiety, indicating good to excellent accuracy. The EPDS-4A also showed good to excellent discrimination, with an AUC of 0.833 for state anxiety and 0.935 for trait anxiety. For the EPDS-3A, the optimal cutoff values were ≥3 for state anxiety (sensitivity 79.2%, specificity 79.7%) and ≥4 for trait anxiety (sensitivity 82.4%, specificity 84.8%), while for the EPDS-4A, the optimal cutoffs were ≥4 (sensitivity 79.2%, specificity 71.2%) and ≥5 (sensitivity 88.2%, specificity 87.9%), respectively. Both reflect strong classification performance. Of note, these thresholds were lower than the cutoff of ≥6 previously reported for the EPDS-3A (46). This discrepancy may be due to differences in the criterion measures used. While the current study employed the STAI, Matthey et al. (46) used DSM-III-R diagnostic criteria for generalized anxiety disorder and panic disorder. The difference in anxiety definitions between the STAI and the DSM-III-R, with the DSM-III-R criteria being more specific to clinical anxiety disorders, may account for the lower cutoff value identified for the EPDS-3A and EPDS-4A in our study compared to that reported for the EPDS-3A in that previous study (46). Further research is needed to evaluate the clinical implications of these findings and to establish optimal cutoffs for both the EPDS-3A and EPDS-4A across diverse cultural populations and with standardized clinical diagnoses.

A higher cutoff score for trait anxiety likely reflects a more stable personality tendency that is less influenced by situational stressors or adverse life changes (47). Trait anxiety is generally less responsive to short-term fluctuations compared to state anxiety (48). In practice, a cutoff of ≥3 on the EPPDS-3A and ≥4 on the EPDS-4A may be useful for identifying acute anxiety triggered by specific stressors, such as childbirth, whereas a cutoff of ≥4 on the EPPDS-3A and ≥5 on the EPDS-4A may help detect more enduring anxious personality traits. The dual application of EPDS-3A and EPDS-4A in evaluating state and trait anxiety provides more nuanced and targeted insights into postpartum anxiety. Trait anxiety reflects a more consistent tendency to respond with anxiety across various situations, in contrast to state anxiety, which is characterized as a more transient and intense emotional state (49). Therefore, higher cutoff values for trait anxiety may reflect its enduring nature, necessitating higher thresholds to differentiate this stable personality feature. Conversely, the sensitivity of state anxiety to transient conditions explains why relatively lower cutoffs may effectively distinguish clinically relevant anxiety episodes from normal daily fluctuations. In addition, a previous study reported that certain demographic and psychosocial characteristics, such as primiparity, partner’s employment, history of depression, unwanted pregnancy, elevated stress levels, family support, and trait anxiety, were associated with state anxiety at six weeks postpartum (50, 51). While these factors may influence the manifestation of state anxiety triggered by childbirth, trait anxiety was identified as a potential predictor. However, further research is needed to clarify the phenotypic differences and the association between state and trait anxiety among postpartum women.

The current findings underscore the equal effectiveness of the EPDS-4A in comparison with EPDS-3A as a practical and accessible tool for screening anxiety in Japanese women at one month postpartum. Depending on the cultural context in which validation has been confirmed, integrating the EPDS-3A or EPDS-4A into routine perinatal care, alongside the full EPDS for depression screening, could enhance the early detection of anxiety symptoms. Clinicians could administer the EPDS-3A or EPDS-4A alongside the full EPDS at the one-month postpartum checkup to identify both depressive and anxiety symptoms during a single clinical visit. The use of any screening tool involves a trade-off between sensitivity and specificity. In the case of the EPDS-3A or EPDS-4A, false positives may lead to unnecessary concern or referrals, while false negatives could result in missed opportunities for early intervention. These implications highlight the importance of follow-up clinical assessments and the careful consideration of cutoff values in practice. Consistent with previous research (17, 51), our findings indicate a significant positive correlation between the anxiety and depression subscales of the EPDS (*r* = 0.691, *P* = 0.0001), supporting the observation that postpartum depression frequently co-occurs with postpartum anxiety and that a history of depression may serve as a risk factor for postpartum anxiety (17, 51). Furthermore, delayed or absent detection of postpartum anxiety may be associated with impaired mother–infant bonding, suboptimal breastfeeding outcomes, delayed cognitive and social development in infants, and an increased risk of severe postpartum depression and other mental disorders in mothers (52–54). Given the high prevalence of postpartum anxiety, its frequent co-morbidity with postpartum depression, and the potential consequences of delayed identification, integrating EPDS-3A or EPDS-4A as a screening tool into routine maternal mental health services in Japan represents an important step toward early detection and intervention.

Despite the demonstrated equal effectiveness of the EPDS-4A in comparison with the widely used EPDS-3A by testing the good fit of the six proposed previous models, which is a key strength of this study, several limitations must be acknowledged. First, the study relied solely on self-reported measures administered at a single time point, without clinician-administered assessments, which are considered the gold standard for clinical diagnosis. This may have led to an overestimation of anxiety prevalence and limited the ability to establish cutoff scores based on clinically diagnosed cases. Additionally, this study assessed only a dataset and did not assess test–retest reliability, leaving the structural and temporal stability of EPDS-anxiety subscales scores unknown. Moreover, qualitative input from the target populations and other measurement tools were not included to support content validity and assess broader divergent validity, respectively. Future research should incorporate diagnostic interviews conducted by trained professionals, qualitative methods, and a wider range of comparator measures to enhance the overall validity of the EPDS-anxiety subscales. Second, although the sample size was sufficient for robust statistical analyses such as CFA and ROC analysis, it was relatively small. Furthermore, healthy participants were recruited from a single obstetric unit of a university hospital in Japan, which may limit the generalizability of the findings in other populations and clinical psychiatry settings. Replication in larger, multi-site cohort studies is recommended to improve external validity. In addition, unlike the EPDS-3A, which has shown its cross-cultural validity across contexts, as the EPDS-4A was newly identified in a Japanese population, future studies are needed to examine its cross-cultural validity. Third, although all participants had full-term deliveries and reported good current maternal health with no complications or negative experiences during childbirth, data were not collected on potentially important variables such as history of depression, educational level, maternal self-efficacy, partner support, or infant behavior. As a result, the observed anxiety levels should be interpreted in light of these limitations, as unmeasured confounding factors may have influenced the results. Moreover, potential sources of bias, such as recall bias and questionnaire order effects, may have inadvertently influenced participants’ responses. Finally, data collection took place during the COVID-19 pandemic, which may have affected participants’ anxiety levels and response patterns. Although all participants were healthy postpartum women exposed to the same contextual conditions, pandemic-related stress could have inflated anxiety scores and thus represents a potential confounding factor. Future studies should aim to validate these findings in non-pandemic contexts to ensure broader applicability.

## 5 Conclusion

This study, based on rigorous psychometric validation, suggests that both the EPDS-3A and EPDS-4A demonstrated good model fit and screening accuracy for postpartum anxiety at one month postpartum. For EPDS-3A, the recommended cutoff scores are ≥3 for state anxiety (sensitivity 79.2%, specificity 79.7%) and ≥4 for trait anxiety (sensitivity 82.4%, specificity 84.8%). For EPDS-4A, the recommended cutoff scores are ≥ 4 for state anxiety (sensitivity 79.2%, specificity 71.2%) and ≥ 5 for trait anxiety (sensitivity 88.2%, specificity 87.9%). Depending on the cultural context in which validation has been confirmed, routine use of the EPDS-3A or EPDS-4A during the postpartum period is recommended to enhance early detection and intervention for maternal anxiety, thereby contributing to improvements in clinical practice and promoting better outcomes in maternal and infant health. Future studies in clinical settings with larger cohorts are warranted to strengthen external validity and confirm the generalizability of these findings.

## Data Availability

The data analyzed in this study is subject to the following licenses/restrictions: The underlying individual-level data cannot be shared publicly, as participant consent for data sharing was not obtained. Requests to access these datasets should be directed to NF, fukui@med.niigata-u.ac.jp.
